# Optimizing the luminescence efficiency of an europium (Eu^3+^) doped SrY_2_O_4_ phosphor for flexible display and lighting applications

**DOI:** 10.1039/d3ra03199c

**Published:** 2023-07-05

**Authors:** Neeraj Verma, Marta Michalska-Domańska, Tirath Ram, Jagjeet Kaur, Abhishek Kumar Misra, Vikas Dubey, Neha Dubey, Kanchan Tiwari, M. C. Rao

**Affiliations:** a Department of Physics, Government Vishwanath Yadav Tamaskar Post Graduate Autonomous College Durg Chhattisgarh India; b Military University of Technology in Warsaw Warsaw Mazovia Poland marta.michalska@wat.edu.pl; c Department of Physics, Bhilai Institute of Technology Raipur Chhattisgarh India jsvikasdubey@gmail.com; d Government Nagarjuna Post Graduate College of Science Raipur Chhattisgarh India; e Department of Physics, Andhra Loyola College Vijayawada Andhra Pradesh India raomc72@gmail.com

## Abstract

This research paper reports the synthesis and luminescence study of an Eu^3+^ activated SrY_2_O_4_ phosphor prepared by a modified solid-state reaction method with varying concentrations of Eu^3+^ ions (0.1–2.5 mol%). X-ray diffraction (XRD) revealed the orthorhombic structure and Fourier transform infrared spectroscopy (FTIR) methods were used to analyse the produced phosphors. Photoluminescence emission and excitation spectra were recorded for varying concentrations of Eu^3+^ ions, and an optimum concentration of 2.0 mol% was found to produce the highest intensity. Under 254 nm excitation the emission peaks were found to be at 580 nm, 590 nm, 611 nm and 619 nm, corresponding to transitions at ^5^D_0_ → ^7^F_0_, ^5^D_0_ → ^7^F_1_, and ^5^D_0_ → ^7^F_2_ respectively. Because of Eu^3+^ inherent luminosity, these emission peaks indicate radiative transitions between excited states of ions, making them useful for developing white light-emitting phosphors for optoelectronic and flexible display applications. The 1931 CIE (*x*, *y*) chromaticity coordinates were calculated from the photoluminescence emission spectra and found to be near white light emission, indicating the potential application of the prepared phosphor for light emitting diodes (white component). TL glow curve analysis was also performed for various concentrations of doping ions and UV exposure times, and a single broad peak was observed at 187 °C. Using the computerised glow curve deconvolution (CGCD) method, kinetic parameters were computed.

## Abstract 2

This research paper reports the synthesis and luminescence study of an Eu^3+^ activated SrY_2_O_4_ phosphor prepared by a modified solid-state reaction method with varying concentrations of Eu^3+^ ions (0.1–2.5 mol%).

## Graphical abstract

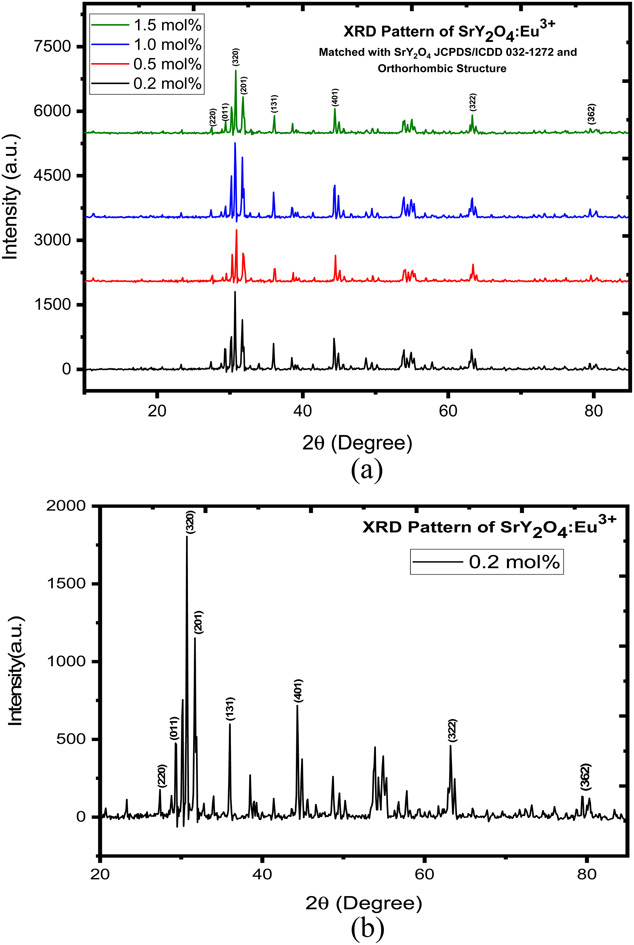

## 1. Introduction

The rosy fantasy of a life that can be controlled with the press of a button and more automation would make the daily lives of humans easier. The current trend of innovation and research has made it easier for people to explore new areas of science and technology. Smart materials are one of the aspects of this new frontier. Smart materials are any substances that are capable of detecting or responding to outside stimuli. There are many other types of stimuli that may be used, including environmental, optical, chemical, electrical, thermal, biological, physical, *etc.*^1–4^ Since the reduction in the size of display systems, luminescent materials have attracted a lot of research attention.^5–7^ Applications for smart luminescent materials are widespread, ranging from polymeric optical fibers and LEDs to displays, self-emitting devices, and light sensors.^8–12^ Achieving durability and adaptability of the electronics and materials used in flexible electronic devices without compromising performance is a critical challenge for smart materials.^13–18^ This obstacle is readily overcome with the use of nanotechnology. The usefulness of several micro- and nanoparticles as smart materials has been reported.^19–22^ Because of this, the intrinsic luminescence feature is the main focus of this study. This property has the potential to be employed in an intelligent (smart) material regarding applications of flexible display.

Europium (Eu^3+^) doped SrY_2_O_4_ is a well-known luminescence material because it exhibits strong and long-lasting red luminescence under ultraviolet or blue light excitation. The luminescence properties of this material are due to the presence of Eu^3+^ ions, which are known for their unique electronic transitions. There are several reasons why Eu^3+^ doped SrY_2_O_4_ is a good luminescence material:

(1) Quantum yield: the quantum yield refers to the efficiency of a phosphor in converting absorbed energy into emitted light. Eu^3+^-doped SrY_2_O_4_ phosphor has a relatively high quantum yield, typically ranging from 60% to 80%. However, it's important to note that the quantum yield can vary depending on the specific manufacturing process and conditions.

(2) Color purity:

• Eu^3+^-doped SrY_2_O_4_: this phosphor typically exhibits a red emission providing good color purity.

• Y_2_O_3_:Eu: this phosphor is known for its red emission, offering high color purity.

• Y_2_O_2_S:Eu: this phosphor generally produces a yellowish-red emission, which also has good color purity.

(3) Cost: the cost of phosphor materials can vary based on several factors, including the availability of raw materials and the complexity of the manufacturing process. Eu^3+^-doped SrY_2_O_4_ phosphor is relatively more expensive compared to some other commonly used phosphors such as cerium-doped yttrium aluminum garnet (YAG:Ce) or various sulfide-based phosphors. The higher cost can be attributed to the specific composition and synthesis methods required to produce SrY_2_O_4_:Eu^3+^ phosphor.

(4) Stability and lifetime: phosphor stability and lifetime are crucial factors to consider in practical applications. Eu^3+^-doped SrY_2_O_4_ phosphor exhibits good thermal and chemical stability, allowing it to withstand the harsh conditions typically encountered in solid-state lighting devices. Additionally, it offers a long operational lifetime, ensuring sustained performance over extended periods.

(5) Excitation wavelength: another important aspect is the excitation wavelength required to activate the phosphor material. Eu^3+^-doped SrY_2_O_4_ phosphor typically requires ultraviolet (UV) or near-UV excitation in the range from 250 to 350 nm. This wavelength range can be achieved using various light sources, including UV LEDs and mercury lamps.

(6) Luminescent properties:

• Eu^3+^-doped SrY_2_O_4_: this phosphor has a relatively high luminescence efficiency, long decay time, and good thermal stability, making it suitable for various applications such as lighting and displays.

• Y_2_O_3_:Eu: phosphor is known for its high luminescence efficiency and good thermal stability, making it commonly used in red-emitting devices.

• Y_2_O_2_S:Eu: phosphor also has high luminescence efficiency and good thermal stability. It is often used in yellowish-red-emitting devices.

(7) Application specifics:

• Eu^3+^-doped SrY_2_O_4_: due to its blue-green emission, this phosphor is often used in applications requiring a specific color range, such as white LEDs and plasma displays.

• Y_2_O_3_:Eu: phosphor finds extensive use in red-emitting devices like fluorescent lamps, plasma displays, and cathode ray tubes (CRTs).

• Y_2_O_2_S:Eu: phosphor is commonly employed in yellowish-red-emitting devices like white LEDs, plasma displays, and CRTs.

Metal oxides with rare earth ion doping have mostly been used for their luminous characteristics. Rare earth ion-doped alkali earth metallic yttrates (MY_2_O_4_, M = Ba, Ca, and Sr) have been seen to exhibit prominent optical emission. According to reports, SrY_2_O_4_:RE^3+^ (RE = Dy, Eu, Tb, Gd, Sm, Ce, and Er) may be made using a variety of techniques, including solution combustion, sol–gel, citrate sol–gel, polyol, oil emulsion, aldo-keto gel, and co-precipitation.^23^ The red optical emission of europium makes it one of the intriguing and flexible lanthanides, making it a prime candidate for luminescence applications. SrY_2_O_4_:Eu^3+^ (SYO:Eu) has a structure similar to CaFe_2_O_4_ and exhibits magnificent down-conversion luminescence. At a wavelength of 611 nm, the very powerful emission line produced by the Eu^3+^ long-lived transition ^5^D_0_ → ^7^F_2_ may be seen. With the excitation of 254 nm (UV region), europium ions are useful for light emitting phosphor and flexible display applications.^24–36^ In this paper, we describe how to easily make new Eu^3+^ doped SrY_2_O_4_ phosphors, which may be utilized to improve the attractive performance of intelligent (smart) materials for flexible displaying applications. SYO:Eu phosphors emit red light when exposed to UV radiation.

## 2. Experimental

The preparation of the SrY_2_O_4_ doped with Eu^3+^ specimen involves a solid-state reaction method. The necessary starting materials, including SrCO_3_, Y_2_O_3_, Eu_2_O_3_, and H_3_BO_3_, Here are the steps for synthesis and characterization of Eu^3+^ doped SrY_2_O_4_ phosphor:

• Preparation of starting materials: SrCO_3_, Y_2_O_3_, and Eu_2_O_3_ are weighed and mixed in stoichiometric amounts to obtain the desired composition of SrY_2_O_4_:Eu^3+^.

• Mixing and grinding: the mixture of powders is then ground together in an agate mortar and pestle for 2 hours to ensure a homogenous mixture.

• Calcination: once the mixing was complete, the resulting powder was placed into a furnace and heated to a temperature of 1000 °C for duration of 1 hour.

• Sintering: after calcination the mixture of powders grind for 1 hour in an agate mortar and pestle and the homogenized powder mixture is then calcined in air at a temperature of 1250 °C for 3 hours to form the desired SrY_2_O_4_:Eu^3+^ phosphor. Higher temperature was needed to ensure that the reaction was complete and that the product was fully formed.

The resulting specimen, SrY_2_O_4_ doped with Eu^3+^, was then allowed to cool to room temperature. The specimen was characterized using various techniques to confirm the desired properties, such as its crystal structure, morphology, and luminescent properties.

## 3. Results and discussion

### 3.1 X-ray analysis

XRD is a widely used technique that involves shining a beam of X-rays onto a sample and measuring the angle and intensity of the diffracted X-rays. The sample's crystal structure and content may be determined from the ensuing XRD pattern. The XRD pattern of Eu^3+^ doped SrY_2_O_4_ phosphors typically shows characteristic peaks corresponding to the crystalline phases present in the sample. The main phase is SrY_2_O_4_, which exhibits aorthorhombic crystal structure. The XRD peaks of SrY_2_O_4_ are sharp and well-defined, indicating a high degree of crystallinity and phase purity. The positions and intensities of the peaks can be used to calculate the lattice parameters and crystal structure of the material.

**Fig. 1:**
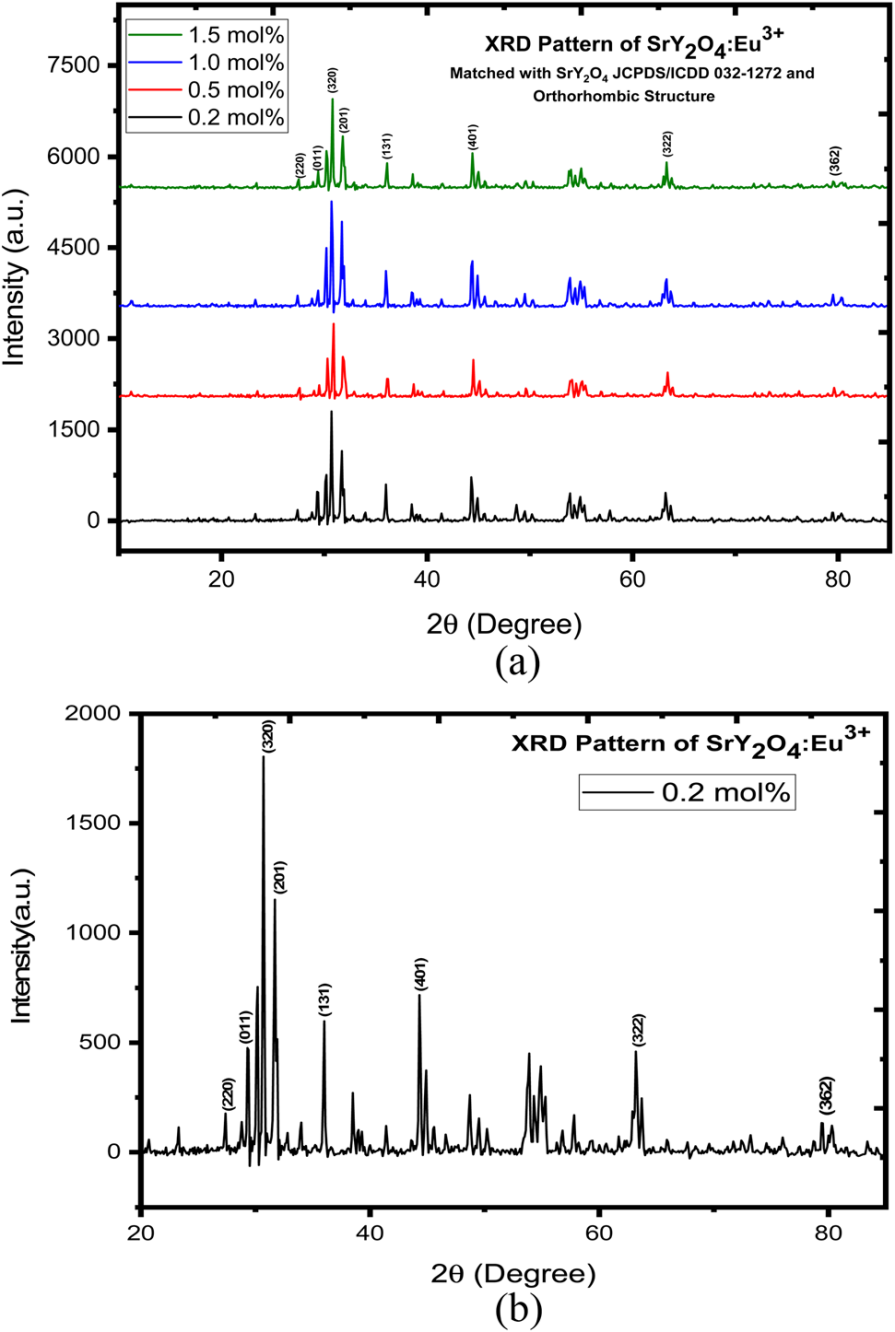
(a) XRD pattern of SrY_2_O_4_:Eu^3+^ phosphor. (b) XRD pattern of SrY_2_O_4_:Eu^3+^phosphor for 0.2 mol%.

The XRD pattern of the sample is shown in Fig. 1 where aorthorhombic structure is displayed. These values match those of the International Centre for Diffraction Data (JCPDS) card No. 032-1272. The crystallite size of the phosphor was calculated using the Scherer equation. The average crystallite size of the phosphor was found to be 34.068 nm (Table 1).

**Table 1:** Crystallite size for 0.2 mol% of SrY_2_O_4_:Eu^3+^ phosphor

*hkl*	Peak position (2*θ*)	FWHM	Crystallite size *D* (nm)	Average size *D* (nm)
220	23.21705	1.38028	5.876894136	34.06808
*011	27.24806	0.13642	59.93173053	
320	30.67829	0.2346	35.12083109	
201	31.62791	0.2148	38.44694105	
131	35.83333	0.13504	61.83951978	
401	44.26357	0.19296	44.45405375	
322	63.17829	0.52107	17.90162841	
362	79.24419	1.14963	8.973048289	

### 3.2 FTIR analysis

**Fig. 2:**
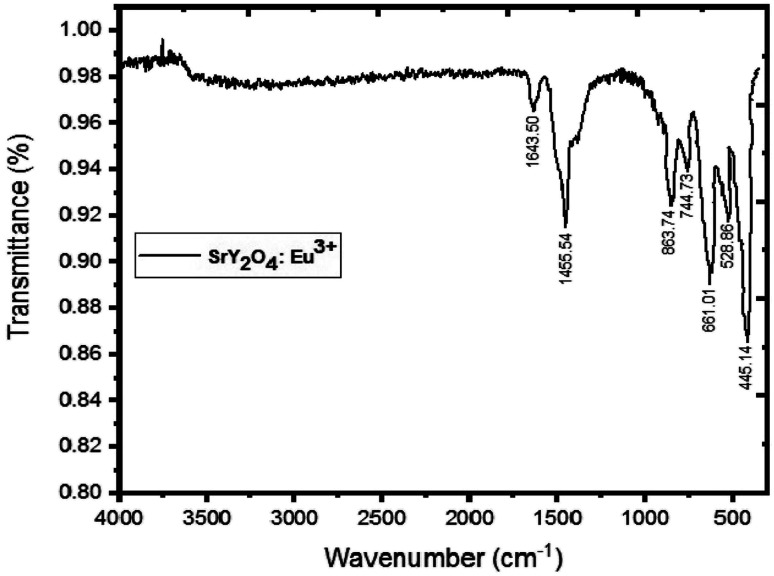
FTIR spectra of SrY_2_O_4_:Eu^3+^ phosphor (2.0 mol%).

Fig. 2 displays the SrY_2_O_4_:Eu^3+^ (2%) phosphor's FTIR spectrum. Strong, sharp peaks may be seen in this spectrum between 445–863 cm^−1^, which are indicative of Y–O vibrations. An IR peak with a wavelength of 1455–1643 cm^−1^ is produced when Sr–O is present.^37^ The production of SrY_2_O_4_ phosphor is confirmed by all of these observed peaks taken together (Table 2).

**Table 2:** Crystallite size for 0.5 mol% of SrY_2_O_4_:Eu^3+^ phosphor

*hkl*	Peak position (2*θ*)	FWHM	Crystallite size *D* (nm)	Average size *D* (nm)
220	23.34254	0.9755	8.317365003	33.4503
*011	27.31909	0.13849	59.04481715	
320	30.8561	0.29096	28.32989183	
201	31.86547	0.26985	30.62172567	
131	36.14804	0.15282	54.69350767	
401	44.49721	0.18065	47.52278414	
322	63.39878	0.3074	30.38083641	
362	79.58315	1.1897	8.692154104	

**Table 3:** Crystallite size for 1.0 mol% of SrY_2_O_4_:Eu^3+^ phosphor

*hkl*	Peak position (2*θ*)	FWHM	Crystallite size *D* (nm)	Average size *D* (nm)
220	23.33911	0.6755	12.01116121	34.7837
*011	27.39439	0.1938	42.20033311	
320	30.7273	0.30141	27.33922054	
201	31.70426	0.28279	29.2088247	
131	36.01264	0.17439	47.91012036	
401	44.35684	0.17304	49.58794225	
322	63.23297	0.58242	16.02063895	
362	79.48164	0.19139	53.9915117	

### 3.3 Scanning electron microscopy (SEM)

Scanning Electron Microscopy (SEM) is a powerful characterization tool that is used to examine the morphology and surface features of europium (Eu^3+^) doped SrY_2_O_4_ phosphors. The technique involves focusing a beam of electrons onto the surface of the sample, causing it to emit secondary electrons that are then collected and analyzed by detectors (Table 4).

**Table 4:** Crystallite size for 1.5 mol% of SrY_2_O_4_:Eu^3+^ phosphor

*hkl*	Peak position (2*θ*)	FWHM	Crystallite size *D* (nm)	Average size *D* (nm)
220	23.35613	0.38574	21.03434538	32.0282
*011	27.56954	0.1934	42.30342242	
320	30.80483	0.28707	28.71023965	
201	31.80803	0.25283	32.67844571	
131	36.17191	0.17999	46.4405259	
401	44.47332	0.19451	44.13273883	
322	63.33333	0.42113	22.16839805	
362	79.5098	0.551	18.75779935	

**Fig. 3:**
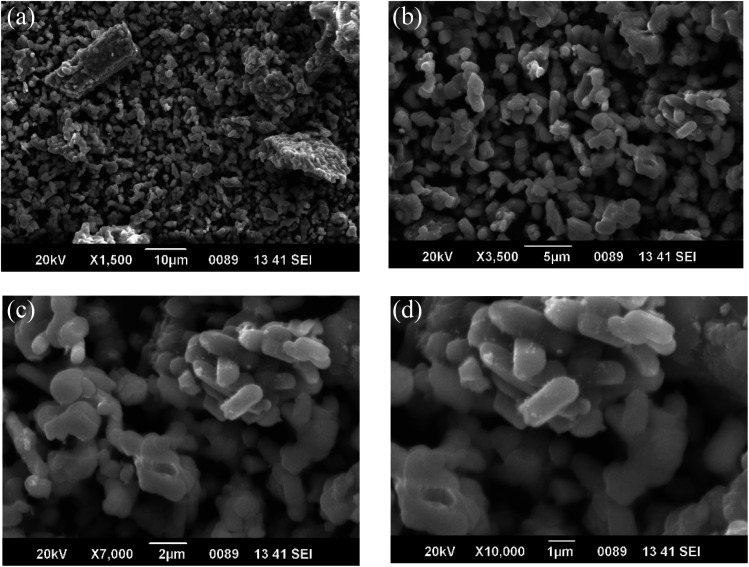
SEM images of SrY_2_O_4_:Eu^3+^ (2.0 mol%) (a) ×1.5k 10 μm (b) ×3.5k 5 μm (c) ×7k 2 μm (d) ×10k 1 μm.

When SEM is used to study Eu^3+^ doped SrY_2_O_4_ phosphors, it provides high-resolution images of the particle size, shape, and distribution (Fig. 3). The technique can also be used to observe any morphological changes that occur as a result of the synthesis method or thermal treatment. SEM imaging can reveal the surface features of the phosphor particles, such as cracks, pores, or other defects, which can affect the luminescence properties of the material (Table 5).

**Table 5:** Average crystallite size for concentration (0.2–1.5) mol% of SrY_2_O_4_:Eu^3+^ phosphor

S. no	Concentration (mol%)	Average crystallite size *D* (nm)
1	0.2	34.068
2	0.5	33.4503
3	1	34.7837
4	1.5	32.0282

### 3.4 Energy dispersive X-ray spectroscopy (EDX)

Energy Dispersive X-ray Spectroscopy (EDX) analysis is a valuable tool for studying the elemental composition of Eu^3+^ doped SrY_2_O_4_ phosphors and can provide important insights into the materials' luminescence properties. By optimizing the elemental composition and concentration of the phosphor, using this technique we can enhance the luminescence efficiency and stability of the material, which can have important implications for applications such as lighting and sensing.

**Fig. 4:**
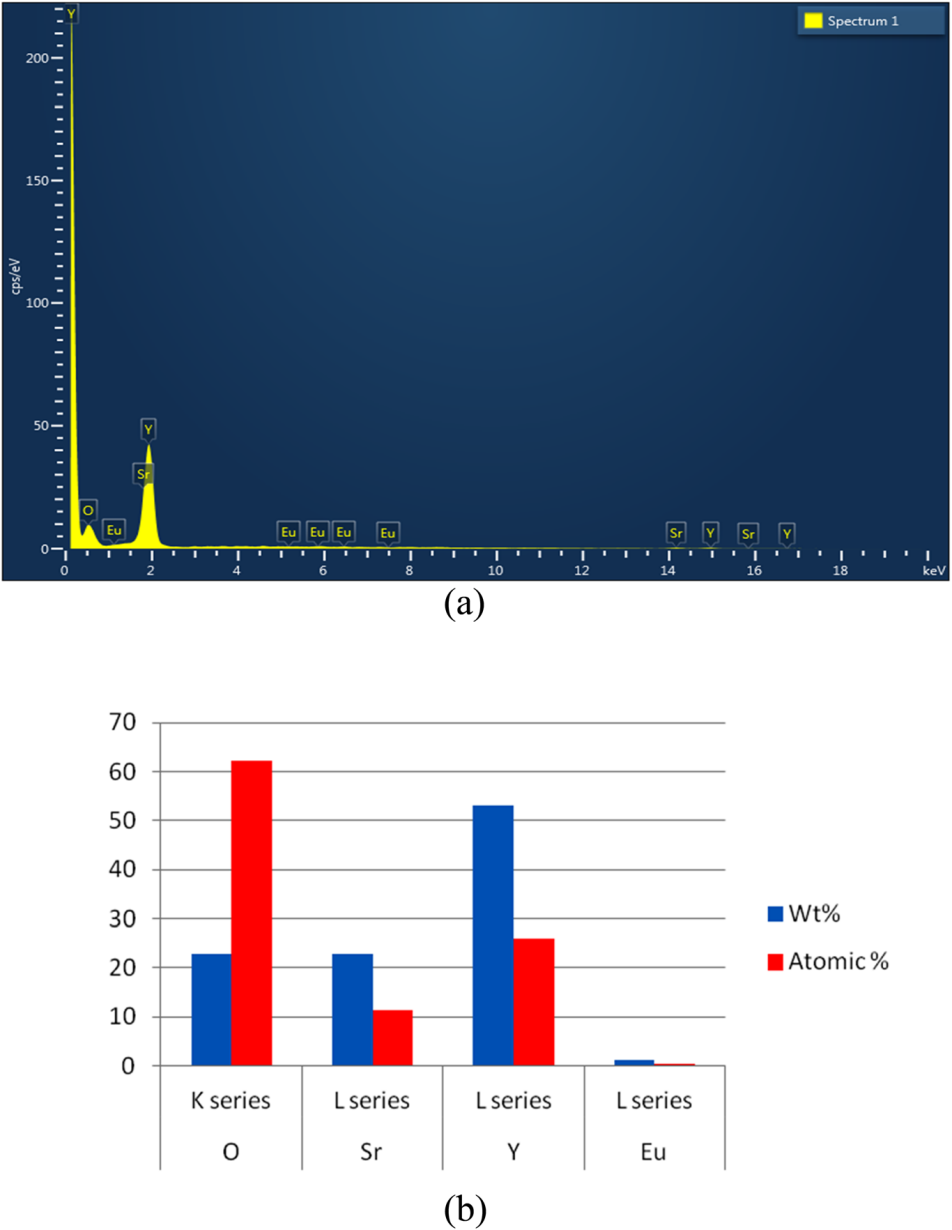
(a) EDX pattern of prepared phosphor Eu^3+^ (2.0 mol%) doped SrY_2_O_4_. (b) Quantitative EDX of Eu^3+^ doped SrY_2_O_4_ phosphor (2.0 mol%).

It is an analysis of the components of produced phosphor. Fig. 4a displays quantitative representations of the sample's Y, Sr, O, and Eu elements. The data supports the conclusion that SrY_2_O_4_:Eu^3+^ phosphor can be synthesized. The qualitative analysis of the process of creating the components is shown in Fig. 4b.

### 3.5 Photoluminescence (PL) observation

**Fig. 5:**
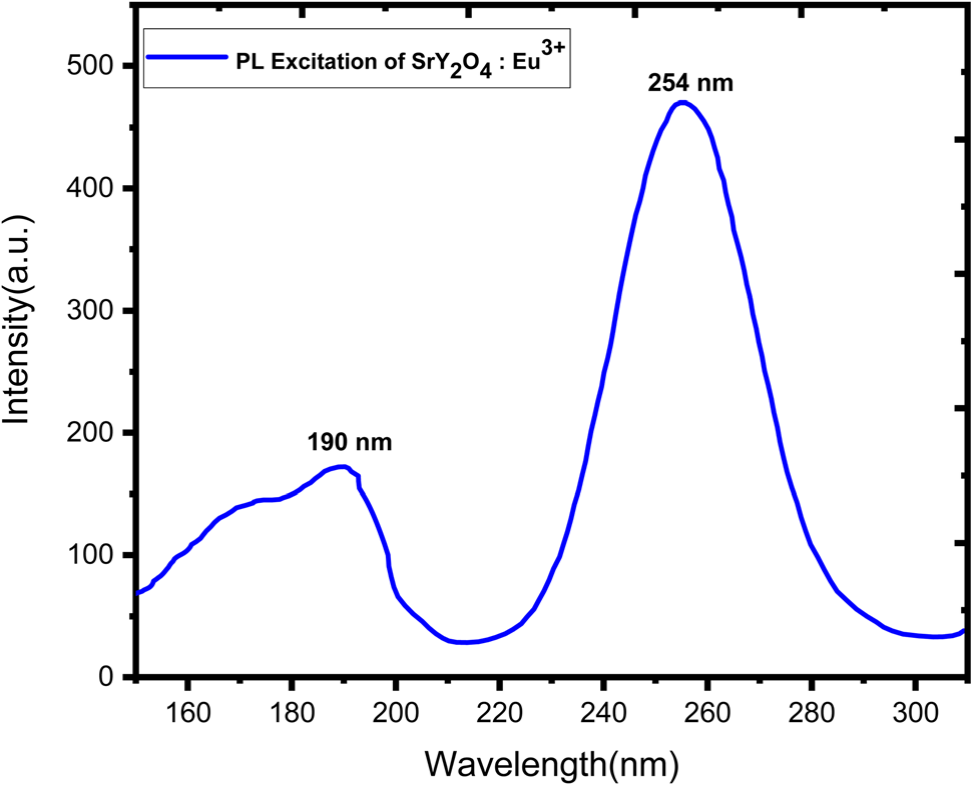
PL excitation spectra of SrY_2_O_4_:Eu^3+^ (2.0 mol%) phosphor.

**Fig. 6:**
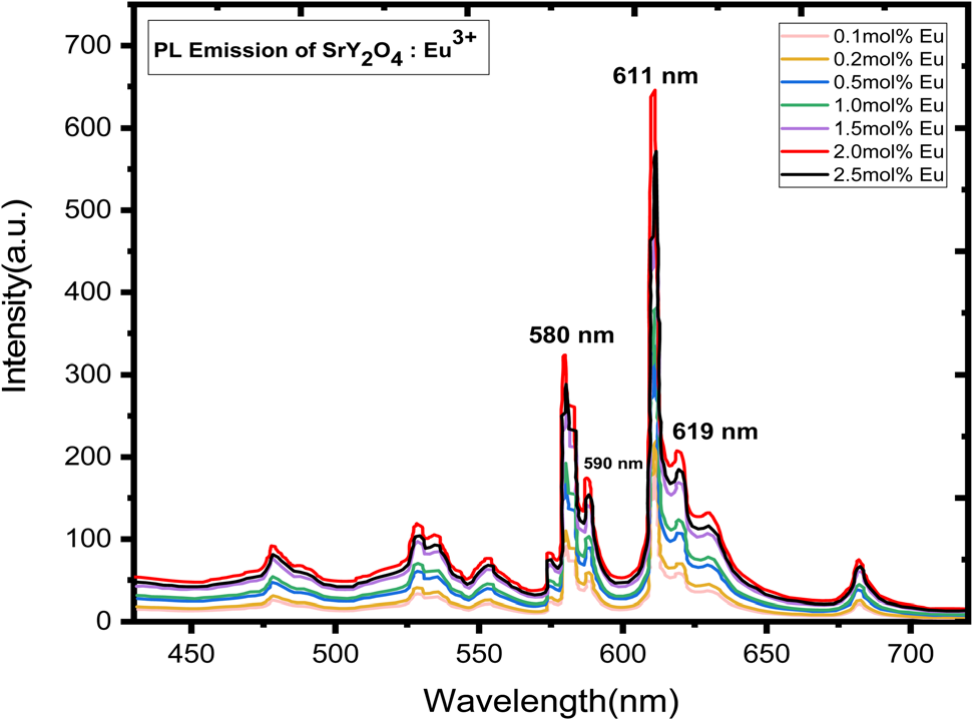
PL emission spectra of SrY_2_O_4_:Eu^3+^ (0.1–2.5 mol%) phosphor.

Doping with Eu^3+^ ions improved the emission spectra of the host SrY_2_O_4_ phosphor, which has no luminescence.^23^ The SrY_2_O_4_ phosphor doped with Eu^3+^ PL excitation spectra are shown in Fig. 5. Excitation spectra were captured at an emission wavelength of 613 nm. It has a wide spectrum between 190 and 254 nanometers. Eu^3+^ doped SrY_2_O_4_ phosphor's PL emission spectrum, measured at 254 nm, shows the typical emissions at 580, 590, 611, and 619 nm. Fig. 6. The emission spectra of Eu^3+^ are produced by transitions between ^5^D_0_ → ^7^F_*J*_ (*J* = 0, 1, 2).^38^ The brightest emission band can be seen at 611 nm, which is associated with the electric dipole transition ^5^D_0_ → ^7^F_2_ (ref. 23–25). The ^5^D_0_ → ^7^F_0_ and ^5^D_0_ → ^7^F_1_ magnetic dipole transitions^60^ give rise to the 580 nm and 590 nm bands, respectively. Due to similar ionic radii, the emergence of the ^5^D_0_ → ^7^F_1_ transition supports the substitution of Eu^3+^ ions at Y^3+^ sites.^39^ The minor separation of the ^5^D_0_ → ^7^F_1_ and ^5^D_0_ → ^7^F_2_ transition lines in the emission spectra of the SrY_2_O_4_:Eu^3+^ phosphor amply demonstrates the considerable influence of the host composition, crystal structure, and coordination environment on luminescence qualities.^40^Fig. 6 shows how the concentration of the dopant affects the intensity of the emission. The intensity continues to rise as the Eu^3+^ ion concentration rises from 0.1 to 2.0 mol%. However, the concentration quenching event causes the intensity to dramatically falldown for 2.5 mol% of the time. Intensity is reduced by quenching, which happens when Eu^3+^ ions move in closer proximity to one another and interact with one another to transfer charge. Eu^3+^ doped SrY_2_O_4_ phosphors using 2.0 mol% had the highest emission intensity, making it suitable for various light-emitting applications (Table 6).

**Table 6:** Quantum yield values for different concentrations (mol%) of SrY_2_O_4_:Eu^3+^

S. no	Concentration (mol%)	Absorption of photons	Emission of photons	Quantum yield
1	0.1	22 988.91862	5870.677682	0.242601398
2	0.2		7114.49341	0.29400116
3	0.5		10 769.65826	0.445048135
4	1		12 347.99237	0.51027162
5	1.5		16 981.28813	0.701739129
6	2		23 090.70313	0.954206169
7	2.5		18 523.81395	0.765482863

The fluorescence quantum yield (*ϕ*_F_) is a measure of the efficiency of fluorescence, representing the ratio of absorbed photons to emitted photons through fluorescence. It quantifies the likelihood of the excited state being deactivated through fluorescence rather than non-radiative mechanisms. The comparative method developed by Williams *et al.* is considered the most reliable approach for determining (*ϕ*_F_). This method involves utilizing well-characterized standard samples with known (*ϕ*_F_) values, enabling accurate measurement and comparison of fluorescence efficiencies.^62^
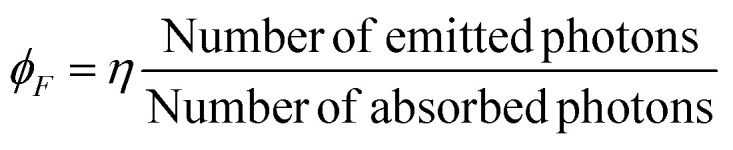
where the *η* denote the refractive index of the sample.

The fluorescence quantum yield (*ϕ*_F_) is a parameter that measures the efficiency of fluorescence by comparing the number of absorbed photons to the number of emitted photons. To determine *ϕ*_F_, one common approach involves analyzing the integrated areas of the absorption and emission spectra. The integrated area represents the number of absorbed photons and the number of emitted photons, respectively. By comparing these values, the fluorescence quantum yield can be accurately calculated (Table 7).

**Table 7:** Table of standard materials and their literature quantum yield values

Compound	Solvent	Literature quantum yield	Emission range/nm	Reference ^63–74^
Cresyl violet	Methanol	0.54	600–650	*J. Phys. Chem.*, 1979, **83**, 696
Rhodamine 101	Ethanol + 0.01% HCI	1.00	600–650	*J. Phys. Chem.*, 1980, **84**, 1871
Quinine sulfate	0.1 M H_2_SO_4_	0.54	400–600	*J. Phys. Chem.*, 1961, **65**, 229
Fluorescein	0.1 M NaOH	0.79	500–600	*J. Am. Chem. Soc.*, 1945, 1099
Norharmane	0.1 M H_2_SO_4_	0.58	400–550	*J. Lumin.*, 1992, **51**, 269–74
Harmane	0.1 M H_2_SO_4_	0.83	400–550	*J. Lumin.*, 1992, **51**, 269–74
Harmine	0.1 M H_2_SO_4_	0.45	400–550	*J. Lumin.*, 1992, **51**, 269–74
2-Methylharmane	0.1 M H_2_SO_4_	0.45	400–550	*J. Lumin.*, 1992, **51**, 269–74
Chlorophyll A	Ether	0.32	600–750	*Trans. Faraday Soc.*, 1957, **53**, 646–55
Zinc phthalocyanine	1% pyridine in toluene	0.30	660–750	*J. Chem. Phys.*, 1971, **55**, 4131
Benzene	Cyclohexane	0.05	270–300	*J. Phys. Chem.*, 1968, **72**, 325
Tryptophan	Water, pH 7.2, 25C	0.14	300–380	*J. Phys. Chem.*, 1970, **74**, 4480
2-Aminopyridine	0.1 M H_2_SO_4_	0.60	315–480	J. Phys. Chem., 1968, 72, 2680
Anthracene	Ethanol	0.27	360–480	*J. Phys. Chem.*, 1961, **65**, 229
9,10-Diphenyl anthracene	Cyclohexane	0.90	400–500	*J. Phys. Chem.*, 1983, **87**, 83

### 3.6 Commission international de I'Eclairage (CIE) coordinates

**Fig. 7:**
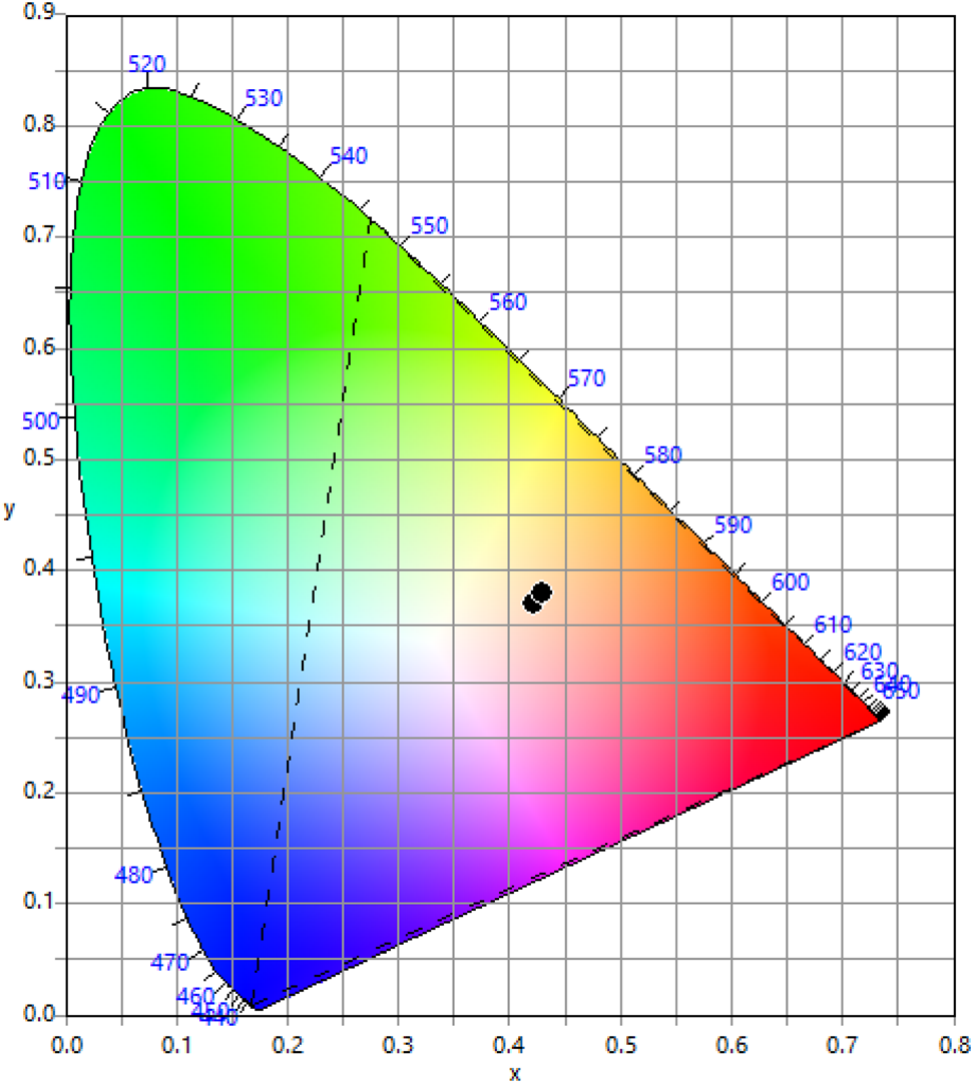
CIE coordinate for SrY_2_O_4_:Eu^3+^ phosphor (0.1–2.5 mol%).

The Commission International de I'Eclairage (CIE) coordinates are a standard way to describe the colour of light emitted by these phosphors. The CIE colour space is a three-dimensional space that describes all possible colours based on three parameters: *X*, *Y*, and *Z*. The *X*, *Y*, and *Z* coordinates represent the relative amounts of red, green, and blue light needed to create a specific colour. The CIE colour space is a useful tool for describing the colour of light emitted by luminescent materials like Eu^3+^ doped SrY_2_O_4_ phosphors. The CIE coordinates of Eu^3+^ doped SrY_2_O_4_ phosphors can be determined using photoluminescence spectroscopy. The emitted light is collected and passed through a monochromator to separate the light into its component wavelengths. The intensity of the light at each wavelength is measured, and the CIE coordinates are calculated based on the spectral distribution of the emitted light. Eu^3+^ doped SrY_2_O_4_ phosphors typically exhibit a near white region luminescence (Fig. 7), with CIE coordinates of according to Table 8. The exact CIE coordinates can vary depending on the specific composition and synthesis conditions of the phosphor.

**Table 8:** CIE coordinates of Eu^3+^ doped SrY_2_O_4_ phosphor with different concentration

Concentration	*x*	*y*	*u*′	*v*′	CCT	CRI
0.1	0.4301	0.3801	0.2568	0.5105	2912	85
0.2	0.43	0.3789	0.2572	0.51	2904	85
0.5	0.4297	0.3797	0.2567	0.5103	2916	85
1	0.4306	0.3802	0.257	0.5106	2906	85
1.5	0.4294	0.3793	0.2566	0.51	2919	85
2	0.4226	0.3692	0.2567	0.5046	2951	85
2.5	0.4295	0.3791	0.2568	0.51	2915	85

### 3.7 TL glow curve analysis

Europium (Eu^3+^) doped SrY_2_O_4_ phosphors exhibit thermoluminescence (TL) properties, which make them useful for radiation dosimetry, medical radiation dosimetry, environmental monitoring, and space radiation dosimetry applications. TL refers to the phenomenon where a material emits light when heated after being exposed to ionizing radiation. The intensity and shape of the TL glow curve, which is the plot of the emitted light intensity as a function of temperature, can provide information about the energy and type of radiation absorbed by the material.

**Fig. 8:**
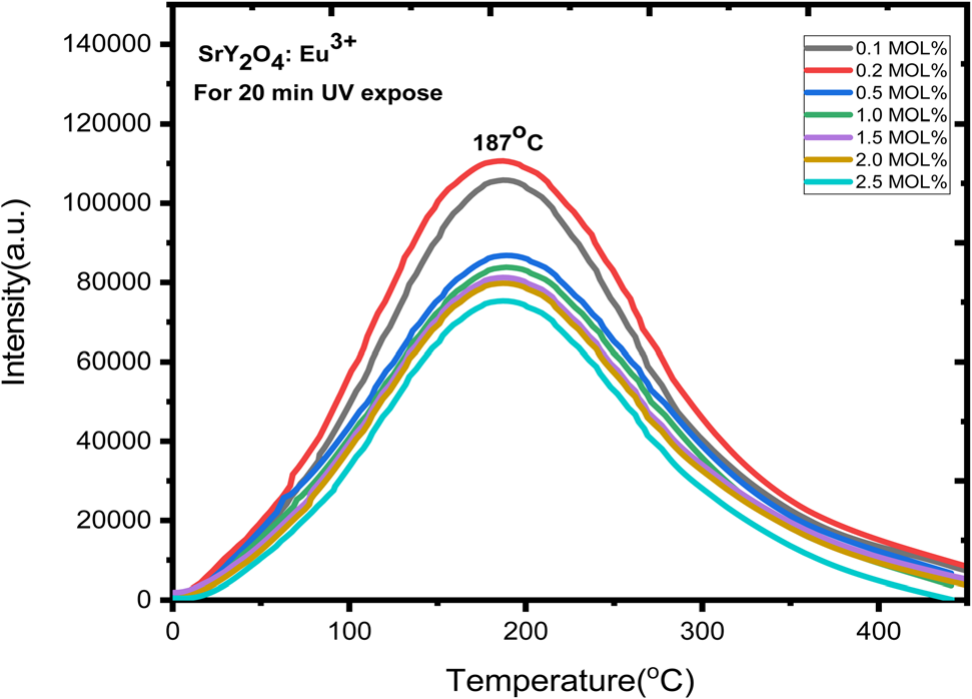
TL glow curve analysis of SrY_2_O_4_:Eu^3+^.

**Fig. 9:**
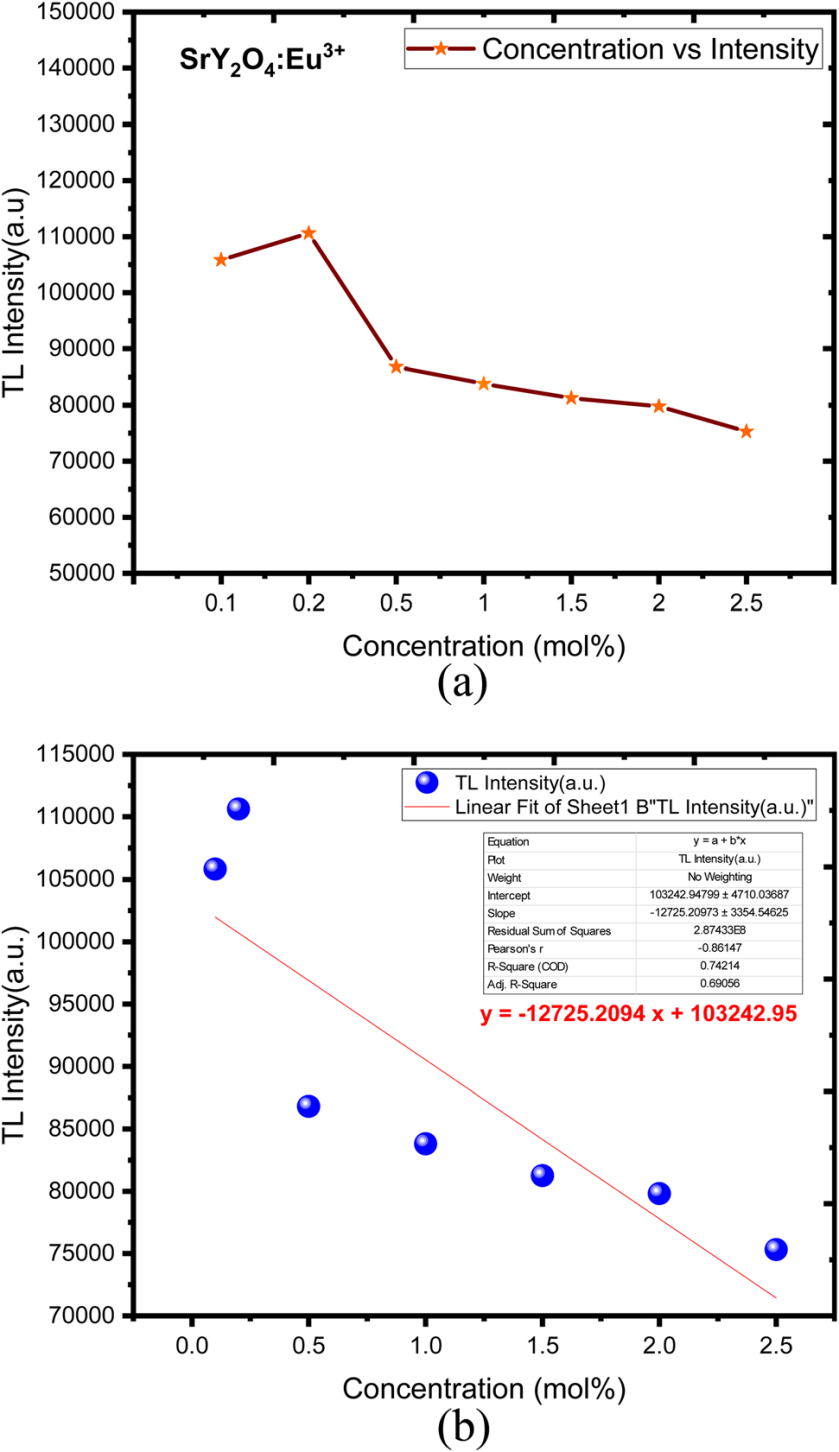
(a) SrY_2_O_4_:Eu^3+^ concentration *vs.* intensity. (b) Linear fit with a mean error bar of SrY_2_O_4_:Eu^3+^ for (0.1–2.5) mol%.

**Fig. 10:**
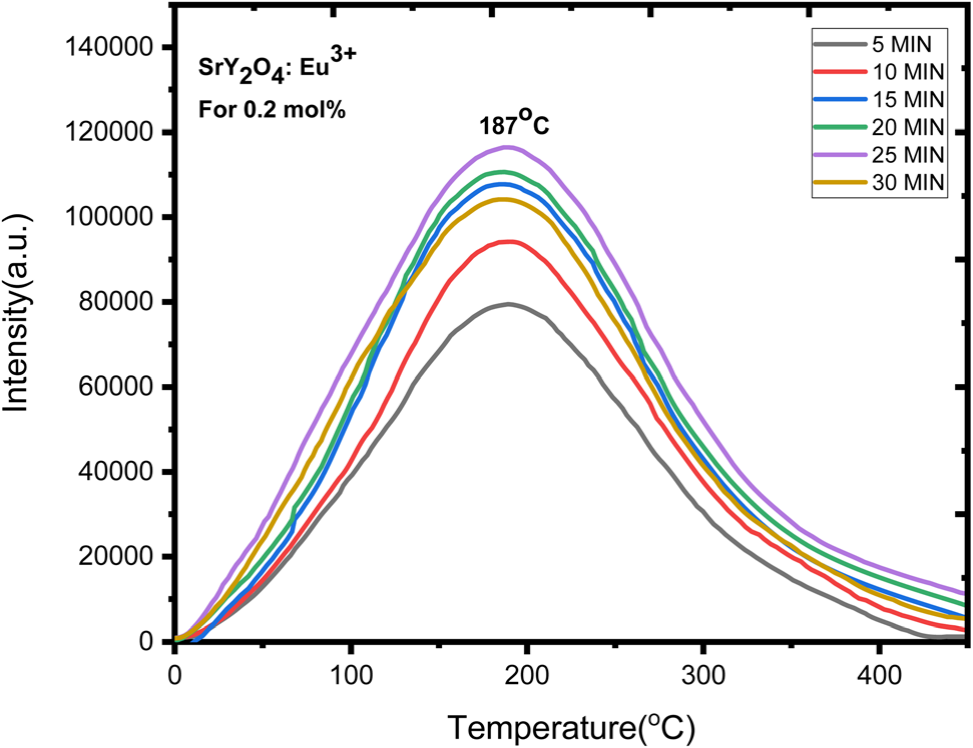
TL glow curve analysis of SrY_2_O_4_:Eu^3+^ for 0.2 mol%.

**Fig. 11:**
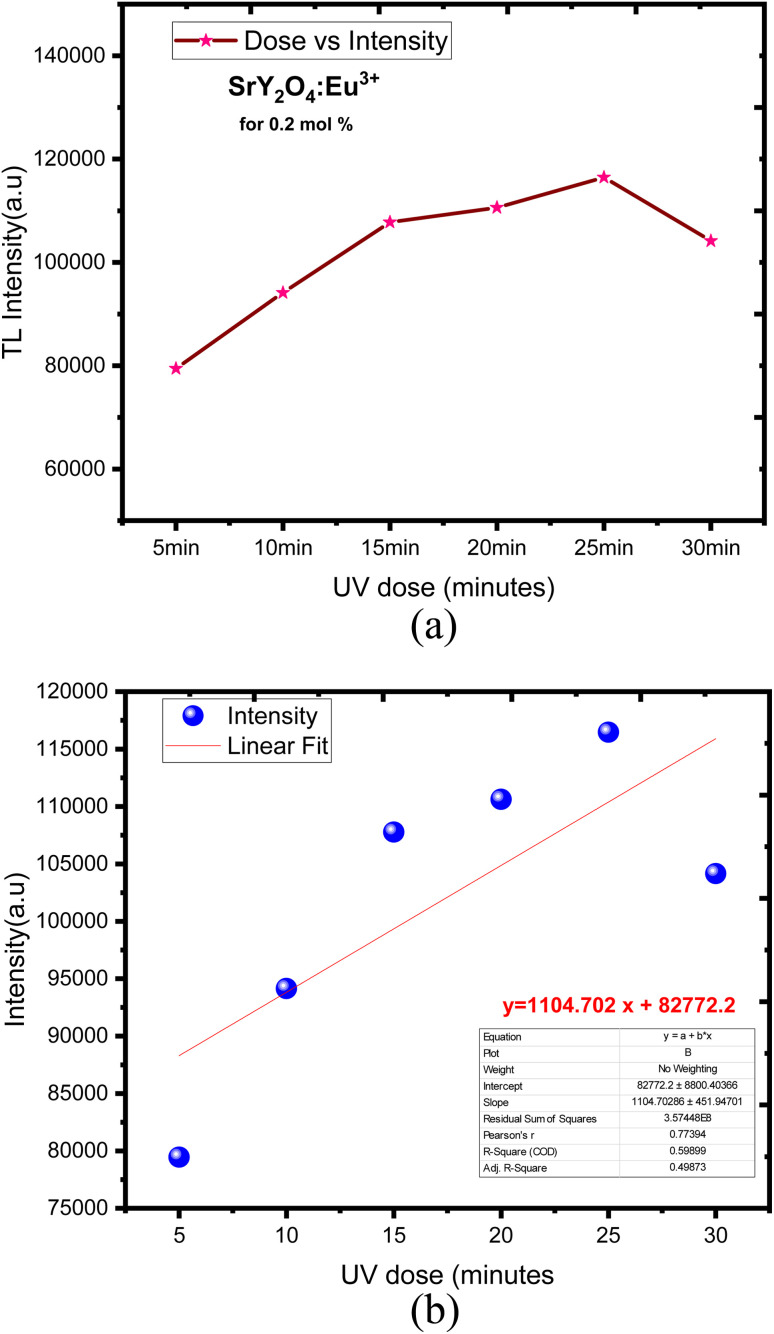
(a) SrY_2_O_4_:Eu^3+^ dose *vs.* intensity. (b) Linear fit with a mean error bar of SrY_2_O_4_:Eu^3+^ for (5–30 min) UV dose.

Europium-doped SrY_2_O_4_ phosphor has a linear response to a 20 minutes UV exposure, as seen by the TL glow curve (Fig. 8). Thermoluminescence (TL) intensity rises with increasing europium concentration (Fig. 8 inset), and its wide peak centers about 187 °C (where the trapped electrons are released from the lattice defects), making it an excellent peak for a thermoluminescence dosimeter. Up to 0.2 mol%, the TL intensity rises; beyond that, it falls as a result of the concentration quenching phenomena (Fig. 9a). The TL glow curve for an optimum concentration of 0.2 mol% is shown in Fig. 10, which displays a linear wide peak with varying UV exposure durations at a fixed rate of heating of 2.5 °C per s. The concentration quenching effect causes TL intensity to drop after the 25 minutes UV treatment for 0.2 mol% (Fig. 11a). Fitting the curve for the optimal concentration of 0.2 mol% using the CGCD method yields an excellent theoretical and experimental fit.

Fig. 9b showed the linear fit corresponding to its mean error with respect to concentration response. The obtained TL phosphor exhibits a linear fit for different concentration over from 0.1 to 2.5 mol%. TL intensity was decreased linearly when the dose was increased, obtaining a determination coefficient *R*^2^ = 0.74214.^75^

Fig. 11b showed the linear fit corresponding to its mean error with respect to dose response. The obtained TL phosphor exhibits a linear fit for different concentration over from 5 to 30 min UV dose exposed. TL intensity was increased linearly when the dose was increased, obtaining a determination coefficient *R*^2^ = 0.59899.^75^

#### 3.7.1 CGCD analysis

**Fig. 12:**
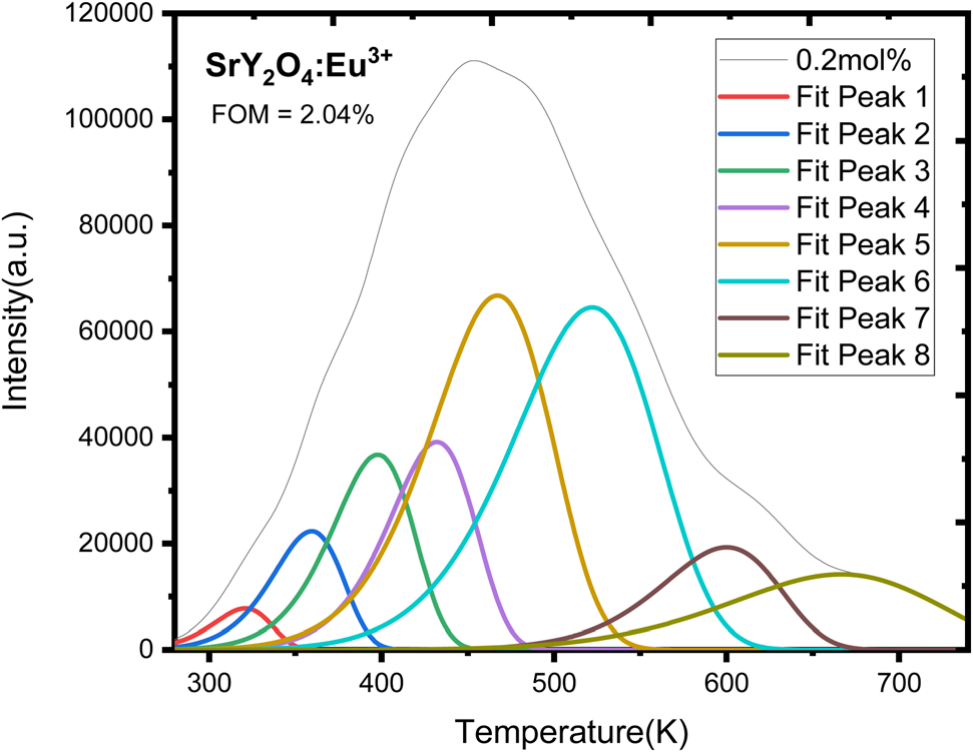
CGCD pattern of UV induced SrY_2_O_4_:Eu^3+^ doped phosphor for optimized UV dose and 0.2 mol% concentration.

TLD phosphor often shows many peaks upon charge carrier (hole or electron) emission. The kinetic factors/parameters (*E*, *b*, and *s*) affect the dosimetric features of TL materials a lot. These parameters will provide significant information regarding the mechanism of phosphor emission. Understanding the kinetic parameters of a TLD phosphor is crucial for producing effective TLDs. An empirical set of equations^41–51^ developed by Chen's allows the estimation of these parameters. Using the Glow fit software, the glow curve is deconvoluted (Fig. 12) in order to implement the peak shape approach. In order to calculate the kinetic parameters of the TL materials' glow peaks, we employed the peak shape approach, also known as Chen's peak method.

Evidently, there are eight wide peaks in the CGCD pattern (Fig. 12), and kinetic parameters are computed for each of those eight peaks in Table 3. After 20 minutes of UV irradiation at varying concentrations of europium ions, the recorded glow curves of the manufactured phosphors reveal a broad peak, suggesting that they are composite in nature. Therefore, the kinetic parameters were deconvoluted and calculated using the CGCD technique. The glow curves of the greatest intensity peak, 0.2 mol% europium ion irradiated with UV for 20 min, were deconvoluted using the CGCD technique using the Glow fit software. The Halperin and Barner formulas provide the mathematical foundation for this computer software. These equations explain the movement of charges between the different energy levels that occur during the process of trap emptying caused by thermal heating. This software calculated the trap level kinetic parameters for each deconvoluted peak. The experimental glow curves were used to fit the theoretically produced glow curves, and the accuracy of the fit was assessed by computing the figure of merit (FOM) associated with each fitting. When FOM values were less than 5%, it was determined that the fits were satisfactory. Current research puts FOM at 2.04%, confirming excellent agreement between theoretically derived and actually observed glow curves.^52,53^Fig. 12 displays the fitted TL glow curves, and Table 3 provides a summary of the CGCD-method-calculated values for the frequency factors (*s*) and trap depths (*E*) of captured charges.

The location of trapping levels inside the forbidden gap is referred to as trap depth or activation energy (E), and it plays a significant role in the loss of dosimetry information that had been preserved in the materials after irradiation. The order of kinetics (b) is the process by which detrapped charge carriers recombine with their equivalents. When the trap is modeled as a potential well, the number of times an electron collides with the wall multiplied by the wall reflection coefficient gives us the frequency factor (*s*). As a result, the trapping parameters of a thermoluminescent material are the foundation for an accurate dosimetry investigation.^54–61^

Here, the information about the trap level obtained from the kinetic parameters (Table 9) shows that almost one electron is trapped there because of the value of (shape factor) 0.435 and it shows the general order kinetics (*b*) and the needed energy to escape one electron from the trap level is high (0.50–0.86 eV), *i.e.* activation energy (*E*), which shows the formation of traps is high and stable. The rate of escaping electrons per second, which falls between 2.12 × 10^9^ and 3.66 × 10^9^ S^−1^, is likewise fairly high.

**Table 9:** Calculation of kinetic parameters using CGCD programme for UV induced SrY_2_O_4_:Eu^3+^ doped phosphor

*T*_1_ (K)	*T*_m_ (K)	*T*_2_ (K)	*τ*	*δ*	*ω*	*μ* = *δ*/*ω*	Activation energy (eV)	Frequency factor s^−1^
298	321	338	23	17	40	0.425	0.5	2.12 × 10^9^
332	359	380	27	21	48	0.4375	0.5175	2.26 × 10^9^
366	398	422	32	24	56	0.4285714	0.5458	2.33 × 10^9^
398	432	458	34	26	60	0.4333333	0.6118	2.65 × 10^9^
420	467	504	47	37	84	0.4404762	0.5011	2.20 × 10^9^
466	522	566	56	44	100	0.44	0.523	2.30 × 10^9^
552	600	636	48	36	84	0.4285714	0.8559	3.66 × 10^9^
587	666	731	79	65	144	0.4513889	0.5823	2.62 × 10^9^

### 3.8 Conclusion

The present study provides valuable insights into the properties of SrY_2_O_4_:Eu^3+^phosphors, as summarized in the following conclusions:

(1) XRD analysis confirmed the existence of a single orthorhombic crystalline phase in the newly synthesized SrY_2_O_4_:Eu^3+^ phosphor, with nanocrystalline behavior.

(2) The SrY_2_O_4_:Eu^3+^ phosphors exhibited a fine surface morphology with a spherical shape which is confirmed by SEM imaging.

(3) FTIR spectra revealed specific vibration modes of Y–O, Sr–O, and the production of SrY_2_O_4_ phosphor is confirmed by all of these observed peaks taken together.

(4) Photoluminescence excitation spectra showed a broad excitation centered at 254 nm, with well-resolved emission peaks 580 nm, 590 nm, 611 nm and 619 nm corresponding to transitions at ^5^D_0_ → ^7^F_0_, ^5^D_0_ → ^7^F_1_, and^5^D_0_ → ^7^F_2_, respectively.

(5) The emission color of SrY_2_O_4_:Eu^3+^ phosphors was in the near white light region, as shown in the CIE 1931 graph, and exhibited good color tenability. These can act as a white light-emitting phosphor for optoelectronic and flexible display applications.

(6) The TL glow curve demonstrated that the thermoluminescence intensity of SrY_2_O_4_:Eu^3+^ phosphors increased with increasing UV exposure time and peaked at 187 °C, indicating a good thermoluminescence dosimeter peak.

(7) The CGCD methodology provided a successful theoretical and experimental fit, with kinetic parameters computed for SrY_2_O_4_:Eu^3+^ phosphor and itshows general order kinetics (*b*) (Table 9).

Eu^3+^ doped SrY_2_O_4_ is a good luminescence material due to its high quantum efficiency, narrow emission bands, long luminescence lifetime, and high stability, making it a versatile material for various applications in optoelectronics, such as solid-state lighting and flat panel displays.

## Conflicts of interest

There are no conflicts to declare.

## Supplementary Material
